# Lateral and Anterior Closing Wedge “9/9” Degree Osteotomy to Correct Both Tibia Vara and Excessive Tibial Slope

**DOI:** 10.1002/atn2.70041

**Published:** 2026-08-06

**Authors:** Julien Druel, Antoine Piercecchi, Chris Wilson, Maher Ghandour, Kristian Kley, Matthieu Ollivier

**Affiliations:** ^1^ Assistance publique des hopitaux de Marseille, France; ^2^ Department of Biomechanics, Institute for Locomotion, Aix Marseille University, CNRS, ISM, Marseille, France; ^3^ Orthopaedic Department Flinders Medical Centre Adelaide South Australia Australia; ^4^ Orthopaedic Care Center Harley Street Specialist Hospital London England

## Abstract

High tibial osteotomy is a key option for patients with unicompartmental overload or instability. Although coronal correction for varus deformity is well established, excessive posterior tibial slope (PTS) is increasingly recognized as a contributor to anterior cruciate ligament graft failure. Conventional strategies often require staged procedures, potentially delaying recovery and compromising bone stock. The “9/9 osteotomy” allows simultaneous correction of varus and excessive PTS in a single stage. The technique combines a lateral closing wedge osteotomy with an anterior retrotubercle closing maneuver. Preoperative planning uses long‐leg standing and lateral radiographs to determine mechanical axis, medial proximal tibial angle, and PTS, which guide wedge dimensions and hinge position. Through a lateral approach, a lateral closing wedge is created toward a posteromedial hinge, followed by removal of an anterior wedge behind the tibial tubercle to decrease PTS. Fixation is performed with a lateral locking plate. The 9/9 osteotomy offers a reproducible single‐stage solution for combined varus and excessive PTS, preserving bone stock and facilitating early rehabilitation.

## Graphical abstract

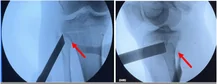

**VIDEO 1:** We first present the case of a patient with excessive tibial slope and postmeniscectomy syndrome, who had undergone more than three ACL reconstructions and remained unstable. The varus correction to be performed was first measured using a Miniaci plan. A first line, the Mikulicz line, and a second virtual line passing through the lateral spine were drawn to define a correction corresponding to 55% of the tibial weight. On calibrated X‐rays, we measured the resection gap required to correct the varus deformity by means of a lateral closing wedge. An 8° correction was planned. A virtual resection wedge of 8° was created, and the height of the osteotomy was measured at the lateral border of the tibia with an oblique cut, preserving a medial hinge throughout the surgery. The tibial slope was then measured using automated software. The tibial plateau was outlined, and the anatomical axis of the tibia was defined proximally and distally along the diaphysis. The slope was measured at 13°, with an intended correction of 8° to restore a normal value of 5°. The software calculated that this 8° correction required 9 mm of closing. We then moved on to the surgery. Clinical examination revealed a positive Lachman test and a negative pivot shift test. A curved incision was performed, starting 1 cm above the joint line and extending distally, allowing visualization of both the tibial tuberosity and the fibular head. The anterior tibial fascia was dissected to expose the bone, and the muscle was detached. The first step was to chisel the proximal tip, which is easily identified through the lateral anatomical approach of the tibia. Once chiseling was completed, the proximal fibular head was released from the tibia, avoiding nerve injury and allowing closure of the lateral wedge. The anterior border of the tibial tuberosity was then approached, with careful subperiosteal dissection of the patellar tendon from the anterior cortex. This allowed a biplanar retrotubercle cut, releasing the distal portion of the tuberosity from the proximal tibia. A small medial window was then created to insert a radiolucent Hohmann retractor from medial to lateral, protecting the neurovascular bundle. This provided safe access for the insertion of K‐wires defining the resection wedge, which were positioned and measured accurately. An oscillating saw was used to complete the osteotomy from anterior to posterior, under the protection of a metallic Hohmann retractor, minimizing the risk of injury. Once the cuts were made, the wedge resection was easily completed, and closure of the lateral cortex was verified to be step‐free. A plate was positioned, and the knee was flexed to assess slope correction. A 4 mm drill was inserted medially into the tibial plateau, acting as a joystick, confirming the anterior closure and slope correction. Once the plateau closed completely anteriorly, full extension was restored to verify stability. A cortical screw was then inserted medially and anteriorly to compress the tibial cortex, while a medial K‐wire was used intraoperatively to confirm correction of the slope during flexion. Here is the final X‐ray of the patient, showing complete correction of both the varus deformity and the tibial slope. Video content can be viewed at https://doi.org/10.1002/atn2.70041.

High tibial osteotomy (HTO) is a well‐established and versatile procedure in knee preservation surgery.^1^, ^2^, ^3^ The most common indication is correction of varus deformity in the coronal plane to delay or avoid the need for total knee arthroplasty (TKA).^4^, ^5^, ^6^, ^7^ Coronal plane malalignment can be corrected using either medial opening wedge (MOW) or lateral closing wedge (LCW) techniques.^8^

Beyond the coronal plane, sagittal plane malalignment can influence knee kinematics,^9^ joint stability,^10^ and ligamentous as well as meniscal loading.^11^ From a biomechanical standpoint, an increased posterior tibial slope (PTS) leads to greater anterior translation of the tibial translation in both anterior cruciate ligament (ACL)‐deficient and ACL‐reconstructed knees, thereby increasing stress on the ACL graft.^12^, ^13^, ^14^, ^15^ A PTS exceeding 12° has been identified as a risk‐factor for graft failure following ACL reconstruction.^16^, ^17^, ^18^, ^19^ Consequently, a slope‐reducing osteotomy using an anterior closing wedge HTO is often considered during revision ACL reconstruction in patients with a PTS > 12°.^20^

Conversely, in patients with posterior cruciate ligament (PCL) insufficiency or PCL graft failure, increasing the PTS through an anterior opening wedge (AOW) HTO may be indicated when the slope is <4°.^11^, ^14^, ^19^, ^21^, ^22^ Preoperative assessment of PTS is typically performed using true lateral knee radiographs with a minimum of 15 cm of proximal tibial shaft visualized, allowing accurate measurement from the medial tibial plateau.^16^, ^23^, ^24^, ^25^, ^26^

However, biplanar deformities involving both coronal and sagittal malalignment remain technically challenging, as they require simultaneous 3‐dimensional correction. Several strategies have been described to address such complex deformities, often necessitating staged procedures.^27^, ^28^, ^29^, ^30^, ^31^

To address this challenge, we describe a simplified single‐stage osteotomy capable of achieving simultaneous correction of both varus and slope deformities to a comparable degree. Based on our experience—where we typically perform approximately 9° valgus correction and 9° of slope reduction—we refer to this technique as the “9/9 osteotomy”.

## SURGICAL TECHNIQUE

### Indications

The lateral and anterior closing wedge (“9/9”) osteotomy is indicated in patients presenting with combined varus malalignment and excessive PTS, particularly when instability or graft failure has occurred following ACL reconstruction.

A symptomatic varus deformity greater than 5° represents a primary indication, especially when accompanied by a steep PTS or anterior tibial translation. According to the ACL + slope tracing risk‐factor algorithm algorithm,^27^ a slope‐reducing osteotomy should be considered when PTS exceeds 13° after ≥2 revision procedures, >15° in first revisions with additional risk enhancers, or >18° to 20° in primary ACL reconstruction. The decision to perform a slope‐correcting osteotomy (SCO) in conjunction with ACL surgery is thus stratified by PTS thresholds and the number of prior reconstructions (Table 1).

**TABLE 1:** Indications for Slope‐Correcting Osteotomy Based on ACL Revision Stage and Tibial Slope

Surgical Stage	Tibial Slope Threshold, °	Indication for SCO
Primary ACLR	>20° without risk factors OR >18° with risk factors	Consider SCO if risk factors present; ACL + LET may suffice
First Revision	>15° with risk factors	Consider SCO
Second Revision	>13°	Strong indication for SCO + ACL + LET
≥Third Revision	>13°	SCO recommended as a salvage surgery

ACL, anterior cruciate ligament; ACLR, anterior cruciate ligament reconstruction; LET, lateral extra‐articular tenodesis; SCO, slope‐correcting osteotomy.

Risk factors that exacerbate anterior tibial translation and increase graft strain—thereby lowering the threshold for slope correction—include medial meniscus deficiency or posterior horn/root tears, static anterior tibial translation ≥5 to 10 mm, prior lateral extra‐articular tenodesis (LET), spontaneous graft rupture, contralateral ACL injury, generalized ligamentous laxity, and participation in pivoting or high‐demand sports. Recurrent graft failure or bucket‐handle meniscal tears further heighten retear risk and support proactive slope management.

Contraindications include severe genu recurvatum (>10°), tunnel malposition necessitating staged reconstruction, prior deep infection, and tricompartmental osteoarthritis.

When appropriately indicated, the 9/9 osteotomy provides a single‐stage solution for simultaneous correction of tibia vara and steep PTS, safeguarding graft integrity and optimizing knee stability in complex primary or revision ACL surgery.

### Preoperative Planning

Comprehensive preoperative planning for HTO requires precise assessment of coronal and sagittal alignment, deformity origin, and joint line orientation.^28^, ^29^ High‐quality, weight‐bearing bilateral long‐leg radiographs remain the gold standard for evaluating lower limb alignment.^30^ Key coronal parameters include the medial proximal tibial angle (normal 85°‐90°) and the mechanical lateral distal femoral angle (normal 84°‐90°).^28^ Joint line obliquity—the angle between the tibial plateau and the horizontal line—should be minimized to <4° to 6° following correction. The joint line convergence angle, ideally between 0° and 3°, reflects intra‐articular deformity and may increase in the presence of chondral wear, meniscal root tears with extrusion, or early osteoarthritis.^31^, ^32^

The authors employ 2‐dimensional preoperative planning following the Miniaci method, targeting the weight‐bearing line at 50% to 55% (occasionally up to 62.5%) of the tibial plateau width. This ensures joint preservation, optimal biomechanics, and minimizes the risk of inducing the opposite deformity through overcorrection^28^ (Table 2).

**TABLE 2:** Summary of Key Parameters

Parameter	Target Value	Notes
MPTA	85° to 90°	Restore mechanical axis to 50% to 55% WBL
mLDFA	84° to 90°	Confirm deformity origin
Target WBL	50% to 55% (≤62.5%)	Avoid valgus overcorrection
PTS target	4° to 6°	Avoid <4° to prevent PCL overload
Hinge position	Posteromedial cortex	Shared hinge for both corrections

MPTA, medial proximal tibial angle; mLDFA, mechanical lateral distal femoral angle; PCL, posterior cruciate ligament; PTS, posterior tibial slope; WBL, weight‐bearing line.

For sagittal alignment assessment, a true weight‐bearing lateral tibial radiograph encompassing at least 15 cm of the proximal tibia is recommended to establish the tibial mechanical axis and accurately measure the PTS from the medial plateau. To enhance reliability and reduce interobserver variability, the “two‐circle method” is preferred.^33^ In this approach, two best‐fit circles—placed at the proximal and distal thirds of the tibial shaft—define the anatomic tibial axis, against which the slope is measured.

A slope‐reducing osteotomy in an ACL‐deficient knee exerts a dual effect: (1) it decreases the PTS and (2) it reduces static anterior tibial translation (SATT), defined as the anterior displacement of the proximal tibia relative to the femur in the sagittal plane. A postoperative PTS of 4° to 6° is considered optimal, balancing reduced graft stress while avoiding PCL overtensioning.^34^ Undercorrection may inadequately reduce SATT, whereas overcorrection (PTS <4°) could theoretically increase PCL strain, though its clinical impact remains uncertain^21^, ^34^ (Table 2).

Planning for a biplanar osteotomy requires careful understanding of the 3‐dimensional hinge position.^35^ During LCW‐HTO, hinge position and axis rotation are key determinants of unintentional sagittal changes, but they can also be leveraged to achieve deliberate, simultaneous correction of both planes.^36^, ^37^ Previous biomechanical and radiographic studies consistently show that MOW HTO tends to increase PTS, whereas LCW‐HTO decreases it.^3^, ^36^, ^38^, ^39^ These changes are most pronounced with large angular corrections or imprecise hinge orientation^36^, ^38^ (Table 3).

**TABLE 3:** Pearls and Pitfalls of Critical Surgical Steps

Surgical Step	Pearls	Pitfalls
Preoperative planning and deformity analysis	Perform meticulous preoperative planning using long‐leg standing and true lateral radiographs to define coronal and sagittal correction targets	Inadequate preoperative planning may result in over‐ or undercorrection in one or both planes
Posteromedial hinge positioning and protection	Position the hinge at the posteromedial cortex and protect it with a medial K‐wire to reduce hinge fracture risk	Insufficient hinge protection may lead to medial hinge fracture or loss of correction
Proximal tibiofibular joint disarticulation	Disarticulate the proximal tibiofibular joint to facilitate lateral wedge closure and reduce lateral cortical stress	Failure to release fibular constraints may compromise wedge closure and increase hinge stress
Coronal and sagittal guidewire placement	Orient K‐wires precisely in both coronal and sagittal planes under fluoroscopic control to guide accurate wedge resection	Mispositioned guidewires can result in unintended slope change or asymmetric closure
Fluoroscopic verification of correction	Use intraoperative fluoroscopy in anteroposterior and true lateral views to confirm correction before final fixation	Lack of systematic fluoroscopic verification may lead to unnoticed malalignment
Target posterior tibial slope adjustment	Aim for a postoperative posterior tibial slope of 4° to 6° to optimize ACL biomechanics	Excessive slope reduction (<4°) may theoretically increase PCL strain
Complete wedge closure and stable fixation	Ensure complete anterior and lateral wedge closure before definitive fixation	Incomplete closure may result in residual deformity or delayed union

ACL, anterior cruciate ligament; PCL, posterior cruciate ligament.

In the present technique, the hinge is set at the posteromedial border of the proximal tibia. A protective K‐wire is inserted at this point to preserve the posterior cortical bone behind it, allowing complete osteotomy through the remaining plane and facilitating both anterior and lateral closure.

### Patient Positioning

The patient is positioned supine on a radiolucent operating table, with a lateral thigh post and a bolster placed beneath the distal femur. The knee is flexed to approximately 90° to permit optimal fluoroscopic visualization. A tourniquet is applied but inflated only after surgical exposure to minimize soft‐tissue ischemia (Video 1).

### Approach

A lateral curved skin incision is made, beginning approximately 1 cm above the joint line, coursing between the tibial tuberosity and the fibular head, and extending distally about 7 cm along the tibial diaphysis. Dissection is carried through the subcutaneous tissue to the fascia of the anterior compartment muscles. The fascia is incised longitudinally, and the anterior compartment musculature is elevated subperiosteally from the lateral cortex.

### Step 1—Proximal Tibiofibular Joint Disarticulation

**FIGURE 1:**
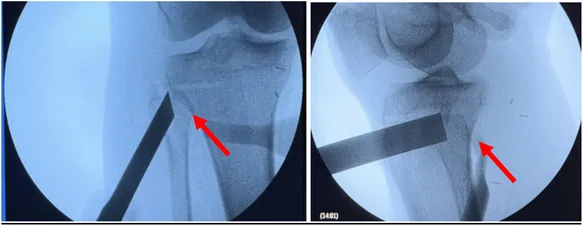
Anteroposterior and lateral fluoroscopic views showing release of the proximal tibiofibular joint using a sharp chisel (red arrow).

The first step involves disarticulation of the proximal tibiofibular joint, which is easily identified via the anterolateral approach. Disarticulation is performed using a sharp chisel, with care taken to avoid injury to the common peroneal nerve. The chisel position is confirmed under fluoroscopic guidance (Figure 1).

### Step 2—Anterior Biplanar Cut

The anterior border of the tibial tuberosity is exposed by carefully releasing the patellar tendon from the anterior cortex. An anterior biplanar osteotomy cut is then performed with an oscillating saw to separate the proximal tibial segment from the distal portion of the tuberosity.

A 3 cm counter‐incision was made medially, through which the sartorial aponeurosis is incised and the posterior surface of the superficial medial collateral ligament gently released. A radiolucent retractor is positioned along the posterior tibial cortex to protect the neurovascular bundle, and a second retractor is placed laterally to shield the soft tissues.

**FIGURE 2:**
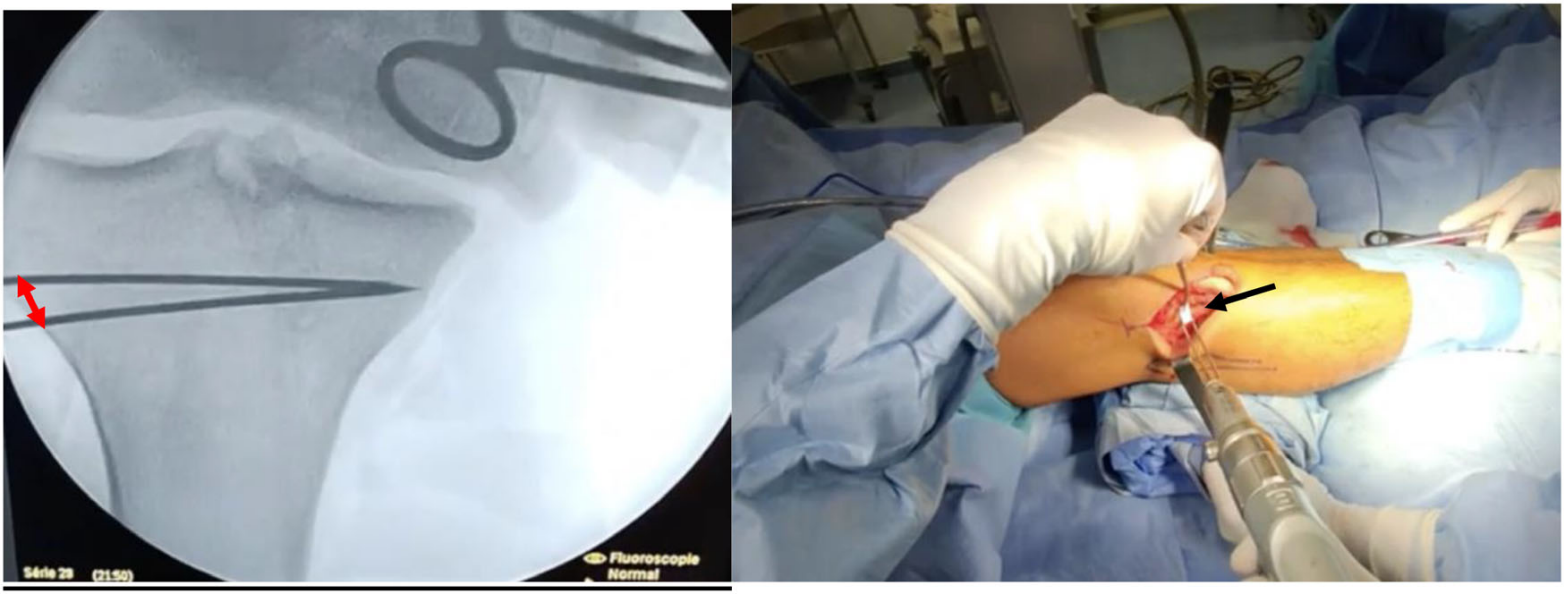
Right knee, patient lying supine. Guidewire positioning for combined coronal and sagittal correction during the lateral and anterior closing wedge (ACW) (“9/9”) osteotomy. Left: Anteroposterior fluoroscopic view of the right knee showing placement of 2 converging guidewires inserted from lateral to medial toward a shared posteromedial hinge. Red arrows indicate the anterior component of the wedge corresponding to posterior tibial slope (PTS) reduction. Right: Intraoperative photograph demonstrating measurement of the inter‐guidewire distance using a calibrated ruler to reproduce the planned angular correction before osteotomy cuts. Black arrow indicates the planned lateral closing wedge for coronal correction of tibia vara.

**FIGURE 3:**
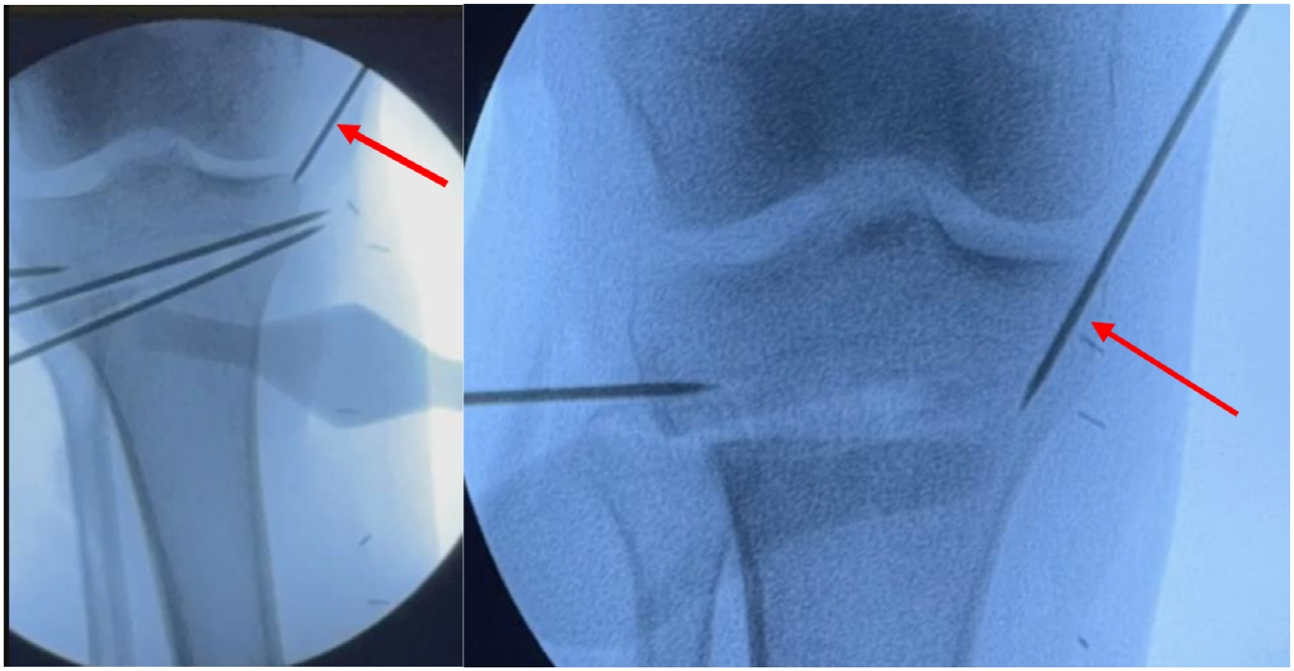
Frontal fluoroscopic cobt. Fluoroscopic view showing placement of the medial K‐wire (red arrows) to protect the medial hinge (left), and fluoroscopic view after completion of the osteotomy with the medial wire safeguarding the hinge (right).

To control PTS correction and protect the posteromedial hinge, guidewire positioning in the sagittal plane is performed according to the preoperative plan. Under fluoroscopic guidance using a true lateral view, two parallel K‐wires are inserted from lateral to medial, converging toward a shared posteromedial hinge located at the posterior third of the medial cortex. The wires are oriented perpendicular to the desired postoperative tibial slope and define the anterior and LCW geometry (Figure 2). This sagittal orientation allows controlled anterior wedge resection posterior to the tibial tubercle, ensuring effective slope reduction while preserving the posterior cortical bone. A protective medial K‐wire may be added to reinforce the hinge and reduce the risk of propagation or fracture during wedge closure (Figure 3). Fluoroscopic verification in both anteroposterior and lateral views is systematically performed to confirm accurate wire orientation and planned correction before completing the osteotomy cuts.

### Step 3—Closure and Fixation

**FIGURE 4:**
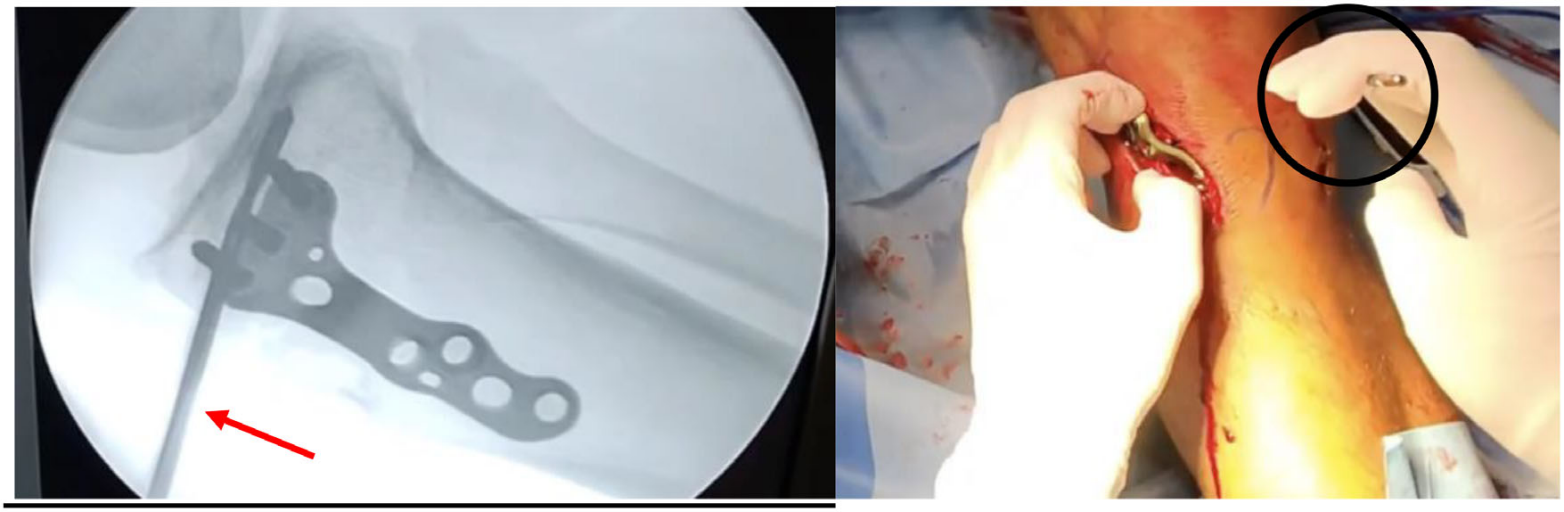
Lateral fluoroscopic control and patient right knee. Anterior wedge closure and control of posterior tibial slope (PTS) during the lateral and anterior closing wedge (ACW) (“9/9”) osteotomy. Left: True lateral fluoroscopic view of the right knee showing insertion of a 4‐mm drill bit into the medial and proximal tibial metaphysis. The red arrow and the black circle highlight the drill bit acting as a joystick to facilitate controlled anterior closure of the osteotomy and reduction of the PTS. Right: Intraoperative photograph demonstrating the drill used as a joystick to achieve complete anterior wedge closure before definitive fixation.

Upon completion of both cuts, the lateral and anterior closures are assessed and confirmed. In particular, fixation is performed using a lateral locking plate system (Active Motion S, Newclip Technics, Haute‐Goulaine, France). The locking plate is positioned under fluoroscopic guidance, and the proximal locking screws are inserted. A true lateral fluoroscopic view is obtained to confirm correction of the PTS. To facilitate complete closure of the anterior wedge, a 4‐mm drill bit is inserted medially into the proximal tibia and used as a joystick (Figure 4).

**FIGURE 5:**
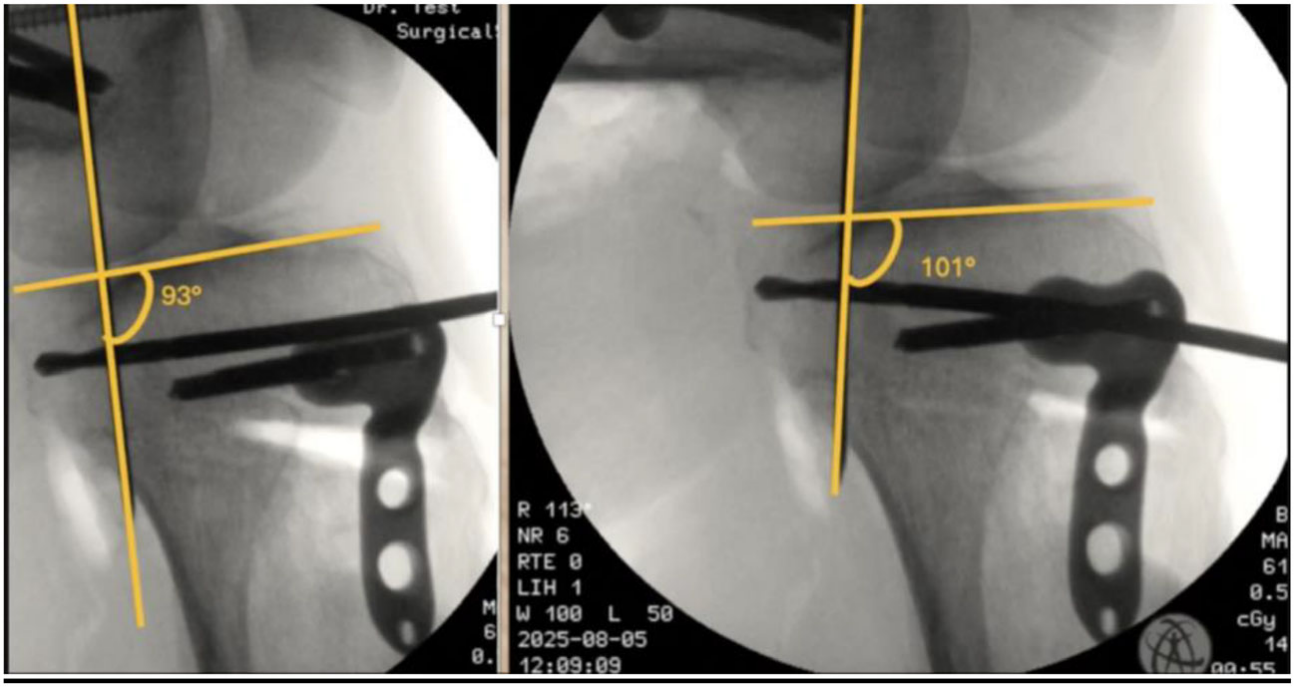
Lateral fluoroscopic views of the knee demonstrating intraoperative verification of posterior tibial slope (PTS) reduction after completion of the anterior closing wedge (ACW) osteotomy.

With the knee extended, the surgeon verifies complete anterior and lateral closure. A cortical screw is then placed through the distal plate hole to achieve medial and anterior compression of the tibial cortex. The remaining locking screws are subsequently inserted, and final coronal and sagittal alignment is confirmed fluoroscopically (Figure 5).

## Postoperative Protocol

Weight‐bearing recommendations are individualized according to concomitant procedures. When no meniscal repair or other protective intervention is required, patients are instructed to maintain approximately 50% partial weight‐bearing with crutches for the first 3 weeks. Thereafter, they progress to full weight‐bearing as tolerated, provided they demonstrated a pain‐free, nonantalgic gait pattern. In cases where a meniscal repair or other procedure requiring protection is performed, weight bearing is appropriately delayed in accordance with the specific rehabilitation requirements.

Passive knee motion (0°‐90°) is initiated on the first postoperative day under the supervision of a physical therapist, emphasizing early quadriceps activation and control of swelling. Active knee extension is restricted for 6 weeks postoperatively to minimize stress on the tibial tubercle and to preserve the integrity of the posterior cortical hinge.

A hinged knee brace locked at −10° of extension is worn during all activities for the first 3 weeks, preventing hyperextension and protecting the osteotomy site. From week three onward, the brace is progressively unlocked to allow 0° to 90° of flexion during ambulation and rehabilitation exercises.

Deep‐vein thrombosis prophylaxis consists of daily subcutaneous heparin injections for 4 weeks, supplemented with graduated compression stockings.

**FIGURE 6:**
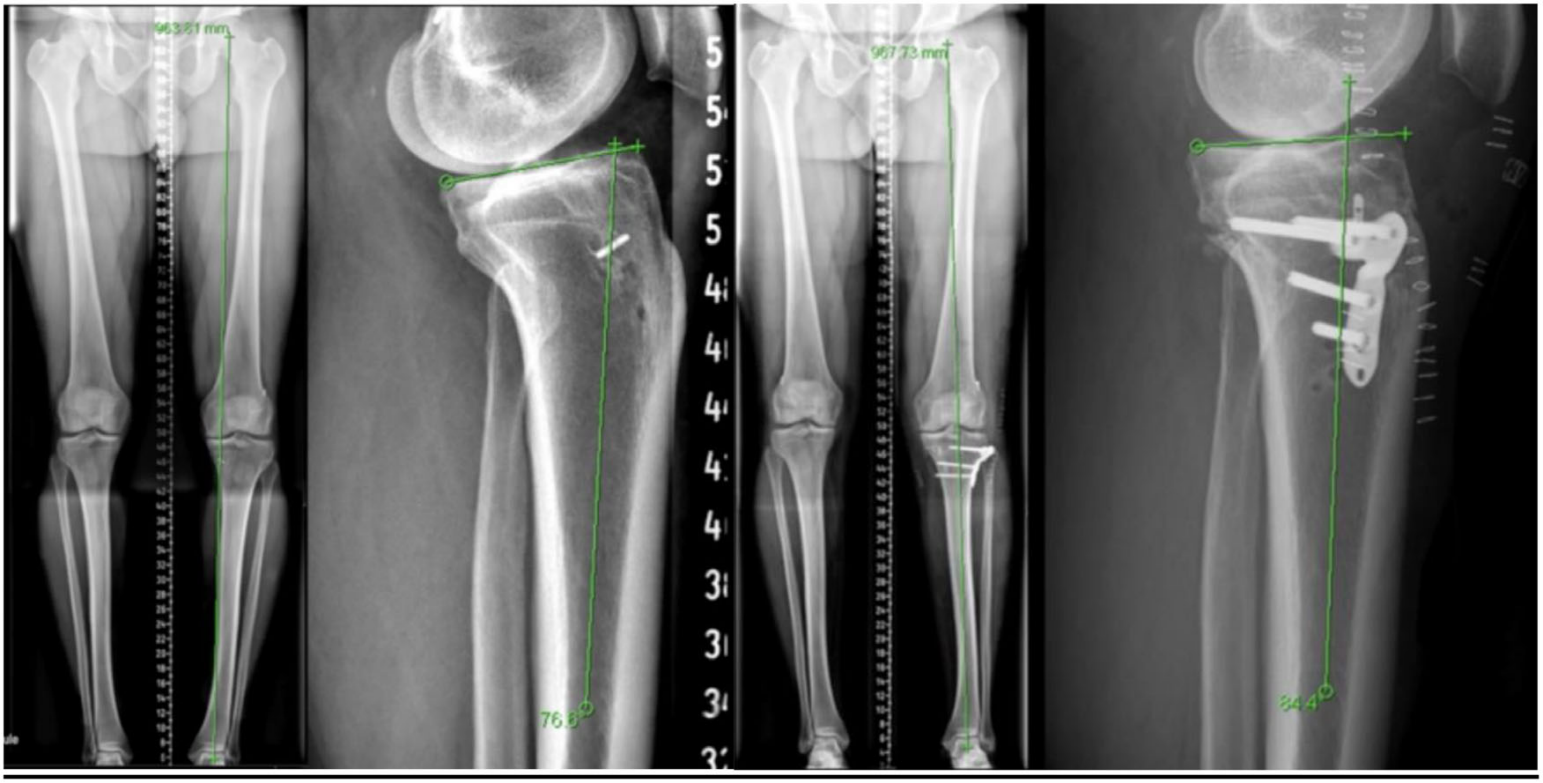
Preoperative anteroposterior and lateral radiographs showing a tibial varus of 7° and a posterior tibial slope (PTS) of 15° (2 images on the left). Postoperative anteroposterior and lateral radiographs after combined lateral and ACW osteotomy demonstrating neutral alignment and a PTS of 6° (2 images on the right).

Wound inspection is performed at 10 to 14 days postoperatively, while follow‐up radiographs at 6 weeks, 3  months, and 6 months are used to evaluate hinge integrity, correction accuracy, and radiographic bone healing (Figure 6).

## DISCUSSION

The present technical note details a reproducible single‐stage technique for simultaneous correction of coronal and sagittal tibial deformities in patients presenting with varus alignment and excessive PTS. By integrating an LCW with an anterior retrotubercle closing maneuver, the “9/9 osteotomy” targets 2 principal biomechanical drivers of ACL insufficiency and graft failure—varus and steep PTS—while preserving bone stock and maintaining patellar height. This approach therefore provides a consolidated strategy in complex primary or revision ACL settings.

Compared with staged procedures or isolated single‐plane corrections, a single‐stage biplanar osteotomy can reduce cumulative surgical morbidity and enable earlier rehabilitation. Our early experience shows reliable correction of the mechanical axis and PTS, with stable fixation that permits early range of motion and progressive weight‐bearing. These observations align with biomechanical and clinical data demonstrating that reducing PTS diminishes SATT and ACL graft loading, thereby improving the biomechanical milieu for graft survival.^20^, ^34^, ^40^

Careful patient selection remains pivotal. The A + STRA framework offers a practical, risk‐stratified guide, indicating that slope‐reducing osteotomy should be strongly considered when PTS exceeds 13° after multiple ACL revisions, >15° in first revisions with risk enhancers, or >18° to 20° in primary ACL reconstruction.^27^ Additionally, recent evidence suggests that lower thresholds (≈10°‐12°) may already confer markedly higher rerupture risk in some populations, reinforcing the value of individualized, risk‐factor informed decision‐making. Risk factors such as medial meniscus deficiency/posterior horn or root tears, SATT ≥5 to 10 mm, prior LET, generalized ligamentous laxity, contralateral ACL injury, or participation in pivoting sports should lower the threshold for intervention.^41^, ^42^, ^43^ Conversely, severe genu recurvatum (>10°), tunnel malposition necessitating staged management, prior deep infection, or tricompartmental osteoarthritis remain relative contraindications.

From a technical standpoint, success relies on meticulous preoperative planning and precise hinge control. Positioning the hinge at the posteromedial cortex, protecting it with a K‐wire, and using fluoroscopy to verify correction in both planes minimizes hinge fracture risk and helps ensure the intended slope reduction.^28^ A retrotubercle cut maintains patellar height and preserves the extensor mechanism, while the LCW provides inherent stability and avoids the PTS‐increasing tendency of MOW HTO.^44^, ^45^

Notwithstanding these advantages, the technique is technically demanding and entails a learning curve. Potential complications include hinge fracture, loss of slope correction or unintended overcorrection (PTS <4°, with theoretical PCL overload), and challenges with closure symmetry in complex, asymmetric deformities (Table 4). Longer‐term, higher‐powered studies are warranted to define union rates, maintenance of alignment, graft survival, and functional outcomes—particularly in high‐demand athletes and multirevision cohorts. Nevertheless, in appropriately selected patients and with careful execution, the 9/9 osteotomy offers a reliable, bone‐preserving, and functionally advantageous solution to restore alignment, optimize knee biomechanics, and protect ligament reconstructions.

**TABLE 4:** Advantages and Disadvantages/Limitations of the Lateral and Anterior Closing Wedge (“9/9”) Osteotomy

Advantages	Disadvantages/Limitations
Simultaneous correction of coronal and sagittal deformities in a single stage	Technically demanding, requires precise preoperative planning
Preserves bone stock compared with medial opening wedge techniques	Risk of hinge fracture, particularly if medial support is insufficient
Maintains patellar height and extensor mechanism integrity	Requires protection of the common peroneal nerve during disarticulation
Provides stable fixation allowing early mobilization and rehabilitation	Steeper learning curve for surgeons unfamiliar with combined‐plane osteotomies
Reduces posterior tibial slope, improving ACL graft biomechanics and stability	Limited indications: contraindicated in severe recurvatum (>10°) or tricompartmental osteoarthritis
Avoids graft‐harvest compromise, suitable for revision ACL surgery	Potential for over‐ or undercorrection if fluoroscopic control and guide placement are inaccurate
Single surgical stage shortens overall recovery compared with staged corrections	Requires careful handling of soft tissues and neurovascular structures
Allows tailored wedge size and correction ratios according to individual anatomy	Postoperative radiographic monitoring is essential to confirm slope and coronal alignment

ACL, anterior cruciate ligament.

In conclusion, the 9/9 osteotomy offers a reliable single‐stage strategy for correcting both coronal malalignment and excessive PTS in appropriately selected patients. By integrating a LCW with an anterior retrotubercle closing maneuver, the procedure preserves bone stock, maintains patellar height, and provides stable fixation, thereby facilitating structured early rehabilitation. When meticulous preoperative planning is combined with strict control of hinge integrity and achievement of slope targets (postoperative PTS ≈4°‐6°), this technique optimizes knee biomechanics, reduces ACL graft stresses, and supports timely return to sport or high‐demand activity. Prospective studies with larger cohorts and long‐term follow‐up are warranted to confirm durability of alignment, union rates, graft survival, and patient‐reported outcomes across diverse populations and revision scenarios.

## DISCLOSURES

The authors (K.K., M.O.) declare the following financial interests/personal relationships which may be considered as potential competing interests: K.K. reports consulting fees for Newclip; payment or honoraria for lectures, presentations, speakers’ bureaus, manuscript writing, or educational events for Newclip. M.O. reports consulting fees for Newclip and Stryker: payment or honoraria for lectures, presentations, speaker bureaus, manuscript writing, or educational events were received from Newclip and Zimmer Biomet. The other authors (J.D., A.P., C.W., M.G.) declare that they have no known competing financial interests or personal relationships that could have appeared to influence the work reported in this paper.
